# Young elite Alpine and Mogul skiers have a higher prevalence of cam morphology than non-athletes

**DOI:** 10.1007/s00167-018-5236-4

**Published:** 2018-10-27

**Authors:** Anna Swärd Aminoff, Cecilia Agnvall, Carl Todd, Páll Jónasson, Olof Thoreson, Mikael Sansone, Leif Swärd, Jon Karlsson, Adad Baranto

**Affiliations:** 1 Department of Orthopaedics, Institute of Clinical Sciences at Sahlgrenska Academy, University of Gothenburg, and Sahlgrenska University Hospital, Gothenburg, Sweden; 2 Sportsmedicine Åre and Åre Ski Academy, Åre, Sweden; 3 Åre Hälsocentral, Box 25, 830 14 Åre, Sweden; 4 Orkuhúsið Orthopedic Clinic, Reykjavik, Iceland

**Keywords:** Femoro-acetabular impingement syndrome (FAIS), Hip joint, Young athletes, Growth zone, Cam morphology, Female athletes, Skiers, Mogul, Alpine

## Abstract

**Purpose:**

To investigate the prevalence of cam morphology in (1) a group of young elite Mogul and Alpine skiers compared with non-athletes and (2) between the sexes.

**Method:**

The hip joints of 87 subjects [*n* = 61 young elite skiers (29 females and 32 males) and *n* = 26 non-athletes (17 females and 9 males)] were examined using MRI, for measurements of the presence of cam morphology (*α*-angle ≥ 55).

**Results:**

The skiers had a significantly higher prevalence of cam morphology compared with the non-athletes (49% vs 19%, *p* = 0.009). A significant difference (*p* < 0.001) was also found between females and males, where 22% of the females and 61% of the males had cam morphology. Among the skiers, there was also a significant difference (*p* < 0.001) between the sexes, where 28% of the females and 68% of the males had cam morphology. This difference between the sexes was not found in the non-athletic group. No significant differences were found between Mogul and Alpine skiers.

**Conclusion:**

Young male elite skiers have a higher prevalence of cam morphology of the hips compared with non-athletes.

**Level of evidence:**

II.

## Introduction

**Fig. 1:**
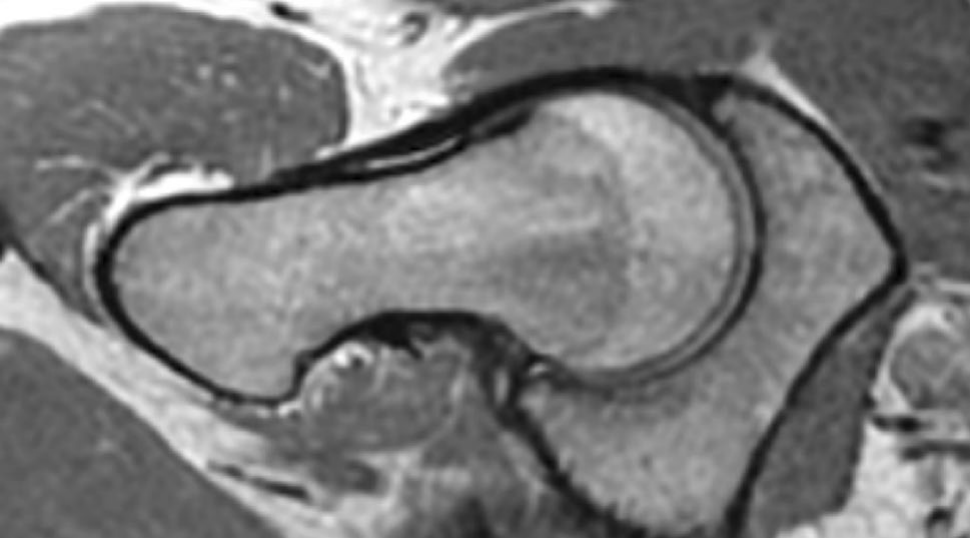
Hip joint with normal morphology of the head–neck junction

**Fig. 2:**
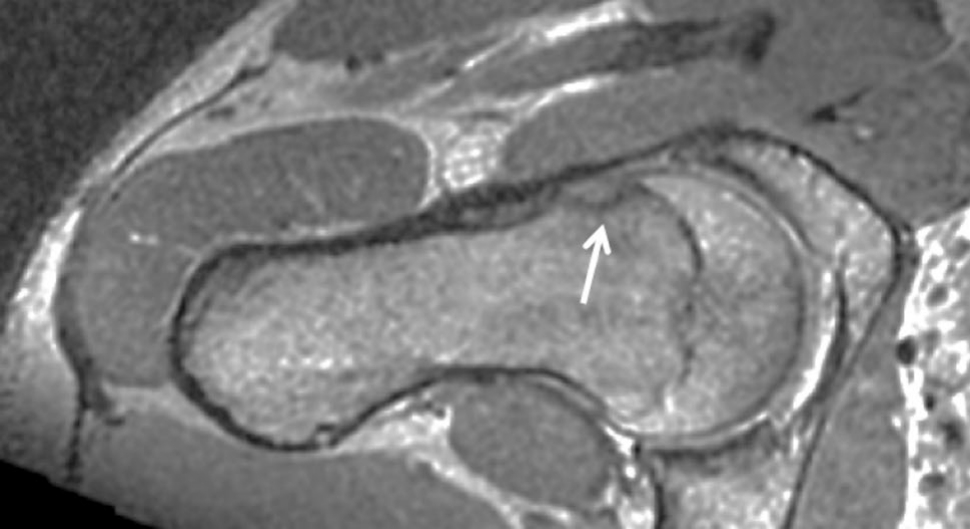
MRI examination of a male skier showing cam morphology with an *α*-angle above > 55° (arrow)

**Fig. 3:**
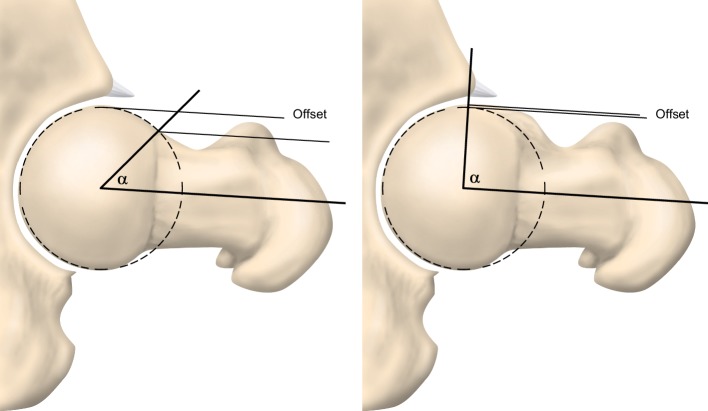
The *α*-angle is used to define the presence of cam morphology and, in this study, a threshold of > 55° has been considered relevant

Femoro-acetabular impingement syndrome (FAIS) is defined as a combination of symptoms, clinical signs and imaging findings of the hip [15, 26, 44]. The abnormal imaging findings can be either femoral based (cam) or acetabular based (pincer) [8, 12, 19, 28]. These two pathologies may occur isolated or in combination [8]. Cam-type FAIS is a condition where the abnormally formed femoral head–neck junction collides with the acetabular margin during flexion and internal rotation of the hip joint (Figs. 1, 2). A measure that quantifies cam morphology is the *α*-angle; the larger the *α*-angle, the larger is the cam morphology, and in previous studies a threshold of > 55° has been considered clinically relevant [6, 29, 38, 39] (Fig. 3). The collision in the hip joint may lead to pain, cartilage injuries of the acetabulum and less frequently to tears of the acetabular labrum [8, 10, 12, 19, 27]. Cam-type FAIS is considered to increase the risk for early osteoarthritis (OA) in the hip joint, while pincer-type FAIS is considered to cause a higher prevalence of labral injuries rather than hip OA [2, 4, 5, 25, 34, 43].

Several studies have shown a higher prevalence of cam morphology to occur in young athletes, in different sports such as soccer, track and field, ice hockey and basketball, and therefore it is hypothesized that the morphology of cam is acquired in relation to vigorous sporting activity during growth [1, 3, 9, 23, 37–39, 42]. Tak et al. [42] found a significant dose–response relationship between the frequency of football practice during skeletal growth and the development of a cam morphology, fortifying the relationship between high-load sporting activity during growth and the development of cam. Agricola et al. [3] showed that young male soccer players gradually develop cam morphology during growth, but after growth plate closure there is no significant increase in the prevalence of cam. Genetics may also play a role in the development of cam, where siblings to patients with cam morphology are at a higher risk of developing the same hip morphology [30]. Furthermore, previous studies have shown that cam morphology is more prevalent among males than females [11]. 

Skiing, both Mogul and Alpine, is a sport that exposes the hip to great forces (high speed and G-forces) in a vulnerable position [18, 22, 41]. There is a constant shift in hip (and knee) flexion from extended to almost maximally flexed during a ski run, which is often on hard and uneven snow. In the Mogul run, there are two acrobatic jumps included, both with high impact on landing.

The hypothesis of this study was that young elite skiers have a higher prevalence of cam morphology compared with a control group of non-athletes, and that cam morphology is more prevalent among male skiers compared to female skiers.

## Materials and methods

### Study population

Seventy-six elite Alpine and Mogul skiers, between 16 and 20 years of age, attending Åre Ski Academy were invited to participate in this prospective study.

To recruit non-athletes, two of the authors visited several high schools and presented the project orally in class. Written information was also distributed. The invited non-athletes were all first-year high school pupils and lived in the same area as the skiers. The criteria defining the non-athletes was that they did not currently nor in the recent past participate in training activities more than two times per week.

Seventy-five skiers and 27 non-athletes agreed to participate in the study.

**Fig. 4:**
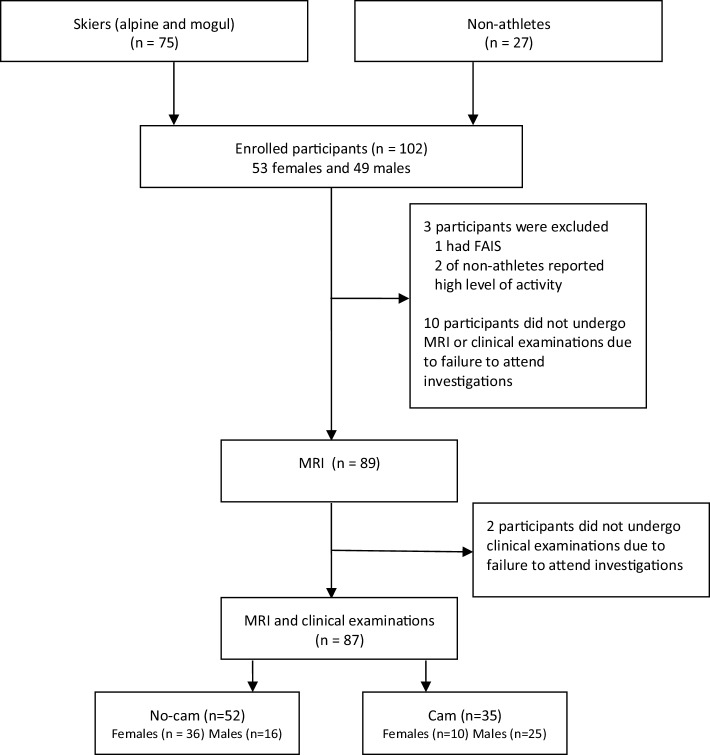
Prisma diagram presenting the participants enrolled in the present study and the MRI results concerning the presence of cam morphology among them

The exclusion criteria for both groups were previously diagnosed hip, spine or pelvic diseases, anomalies and previous surgery to the hips, spine or pelvis (Fig. 4).

Participation was completely voluntary and the participants could withdraw at any time. Written consent was given by all individuals, and for participants younger than 18 years, written consent was also obtained from their parents.

The present study was approved by the Regional Ethical Review Board at the Sahlgrenska Academy, Gothenburg University, Gothenburg, Sweden (ID number: 692-13).

### MRI examination

All subjects underwent MRI of both hips without contrast. The MRI that was used was a GE Optima 450 Wide 1.5T (GE Healthcare Bio-Sciences Corp, Piscataway, NJ, USA), at Östersund Hospital, Sweden. Cor T2 Fat Sat and Ax 3D Cube sequences were obtained, angled to the femoral neck using a coil surface of HD 8 Channel Cardiac Array (GE Healthcare Bio-Sciences Corp).

Evaluation of the MRI images included two parts. First, the status of the growth plate was evaluated as being either closed or open, based on the appearance of the capital femoral growth plate on MRI using the same method as Siebenrock et al. [38]. Second, the *α*-angle was measured. Seven measurements, from 9 o’clock to 3 o’clock (180°), to determine the morphological features of the femoral head–neck junction were performed [19]. Measurement of the *α*-angle was performed according to Nötzli et al. [27]. The *α*-angle was measured between the femoral neck axis and a line from the centre of the femoral head to a point where the contour of the femoral head–neck junction exceeds the radius of the femoral head (Fig. 3). A cam morphology was considered present when the *α*-angle was above or equal to 55° [6, 29, 38, 39].

The *α*-angle and the status of the growth plate were evaluated and measured blindly by a resident radiologist, under the guidance of a senior consultant radiologist. The images were evaluated according to a standardized protocol, including standardized assessment of the *α*-angle and growth plate as previously described. To test the inter-observer reliability, MRI images were selected randomly from ten participants and were re-examined by a senior consultant radiologist.

### Statistical analysis

Data were analysed using IBM SPSS Statistics for Windows (Version 24.0. Armonk, NY: IBM Corp). The description of data was expressed in terms of mean and standard deviation (SD). The inter-rater reliability of the measurements was determined with the intraclass correlation coefficient (ICC 2.1) (two-way random model, absolute agreement, single measures). To categorize the level of agreement among ICC values, we used the classification system proposed by Shrout and Fleiss [36]. ICC values less than 0.40 represent poor, values between 0.40 and 0.75 represent fair to good, and values above 0.75 represent excellent reliability. SEM, a reliability statistic which quantifies measurement error in the same units as the original measurement was calculated as SEM = SD $\surd {\text{E}}1 - {\text{ICC}}$, where SD is the standard deviation of the difference between observations. All tests were two-sided and significance was set at *p* < 0.05 for each test. A Pearson Chi square test was performed to evaluate the distribution of cam between the sexes.

All pupils of Åre Ski Academy were invited to participate in the present study and comprised the study population; therefore no power analysis was calculated prior to the study. The intention was to match each skier with a control, but it was difficult to recruit accurate age-matched non-athletes. Therefore, the groups were not matched.

## Results

Table 1 presents the study population and the distribution of cam morphology between the groups. Seventy-five skiers agreed to participate, 35 females and 40 males (Fig. 4). Twenty-seven non-athletes, 18 females and 9 males, all 16 years old agreed to participate. Three subjects were withdrawn from the study as they did not fulfil all inclusion criteria. Also, failure to attend investigations made only MRI data from 87 participants available for the final analysis.

**Table 1:** Distribution of cam morphology between the groups

	Subjects (*n* = 87)	Cam (*n* = 35)	No cam (*n* = 52)	*p* value
Age (years)	17.7 (1.3)	18.1 (1.2)	17.4 (1.3)	0.019
Skiers	61 (70.1%)	30 (49.2%)	31 (50.8%)	0.009*
Controls	26 (29.9%)	5 (19.2%)	21 (80.8%)	
Female	46 (53%)	10 (22%)	36 (78%)	< 0.001*
Male	41 (47%)	25 (61%)	16 (39%)	
Female skiers	29 (47.5%)	8 (27.6%)	21 (72.4%)	< 0.001*
Male skiers	32 (52.5%)	22 (68.8%)	10 (31.3%)	
Female controls	17 (65.4%)	2 (11.7%)	15 (88.2%)	ns
Male controls	9 (34.6%)	3 (33.3%)	6 (66.7%)	
Alpine	45 (73.8%)	19 (42.2%)	26 (57.8%)	0.068*
Mogul	16 (26.2%)	11 (68.8%)	5 (31.3%)	

Values are mean and (SD)

*Chi-squared test, prevalence of cam between groups

### MRI

The result of the inter-observer test (ICC) analysis for the *α*-angle [ICC 0.75 (SEM 1.8)] indicated a good level of agreement.

All participants had MRI-verified closed growth plates.

Out of the seven measurements (from 9 o’clock to 3 o’clock), the highest *α*-angle was generally measured at 1 o’clock in the antero-superior region of the femoral head–neck junction. In 87 participants, 174 hips were analysed, where a total of 53 hips (30%) had cam morphology. The measurements of the *α*-angle showed equivalent results for the right and left hips, with 26 hips (49%) on the right side and 27 hips (51%) on the left side.

At an individual level, 35 of the subjects (40%) had cam morphology: 10 females (22%) and 25 males (61%) (Table 1). Eighteen subjects of the whole population (21%) and 17 of the skiers (28%) had bilateral cam morphology.

The skiers had a significantly higher prevalence of cam morphology compared with the non-athletes (*p* = 0.009), where 30 of the skiers (49%) and only 5 of the non-athletes (19%) had cam (Table 1). No significant difference was found between the Mogul and Alpine skiers.

A significant difference was found between the sexes in this study population, where 10 of the females (22%) and 25 of the males (61%) had cam morphology (*p* < 0.001). Among the skiers there was also a significant difference between females and males (*p* < 0.001), where 8 of the females (28%) and 22 of the males (69%) had cam. This difference between the sexes was not found in the non-athletic group.

Across the sample, even the participants with an *α*-angle less than 55°, a tendency was shown with the skiers having higher *α*-angle values in all measurements (from 9 o’clock to 3 o’clock) compared with the control group.

## Discussion

The most important findings of the present study were that (1) a significantly higher prevalence of cam morphology was shown amongst the young skiers compared with the non-athletes (49% vs. 19%), and (2) there was a significantly lower prevalence of cam amongst the female subjects compared with their male counterparts (22% vs. 61%).

The exact mechanism behind the formation of cam has still not been identified. Siebenrock et al. [37] suggest that cam morphology is a consequence of an alteration of the growth plate (extension of the growth plate) and that this is more common in young athletes. It is possible that the cam morphology is acquired, due to repetitive and heavy load, during the years associated with the adolescent growth spurt, because of a vulnerability of the skeleton and growth plate. Bailey et al. [7] showed that there is a significant time difference in peak bone velocity (bone mineral content accumulation) between girls (12.5 years) and boys (14.1 years). The peak height velocity appeared approximately 1 year earlier than the peak bone velocity (girls 11.8 and boys 13.5 years). Because of the lag time between the peak bone height velocity and the peak bone mineral content alongside higher hormonal levels, the skeleton is especially more responsive to mechanical stimuli during the years accompanying the adolescent growth spurt [7]. Biomechanical studies have shown that the growth plate is the most vulnerable part in the growing hip and is especially sensitive to heavy loading during hip flexion and/or external rotation [20, 33]. The growth disturbance may be due to repetitive micro-fractures, which could disrupt the arterial blood supply to the growth plate and inhibit the normal ossification of the chondrocytes, resulting in a delayed closure of the growth plate [24, 35], and the development of cam morphology.

The prevalence of cam morphology may vary depending on imaging modality, definition of cam morphology, age and sex. In the present study, we chose MRI because it has been used in several previous studies investigating cam, and cam morphology was considered present when the *α*-angle was above or equal to 55° [6, 19, 27, 29, 38, 39]. All participants in the present study had closed growth plates and were therefore considered skeletally mature [3, 29]. In the elite skiers group, there was an equal distribution between the sexes, but in the control group there was an overrepresentation of females.

Compared with other studies, with regard to asymptomatic controls, the present study showed a slightly higher prevalence of cam in both females and males. Others have reported a prevalence of cam morphology in asymptomatic study populations ranging from 5.2 to 24.7% (females 5.2–5.4% and males 9–24.7%) [1, 14, 16, 32, 38]. In the present study, the prevalence was 19.2% (females 11.7% and males 33.3%). The control group was selected from the same geographical area as the Åre Ski Academy is located, and therefore they might be more active than average high-school pupils, as the population in this region is generally active. Moreover, it was difficult to recruit controls and the smaller cohort size, compared with the skiers, might have affected the results. Especially the male controls are underrepresented in this study and this might have contributed to the relatively high prevalence of cam among the controls.

Moreover, the present results correlate well with those in previous studies involving athletes in different sports such as soccer, track and field, ice hockey and basketball [1, 3, 9, 23, 29, 37–39, 42]. Philippon et al. [29] found that the prevalence of MRI-verified cam morphology (*α*-angle ≥ 55°) was higher among young skiers (40%) than the average non-athletic control (as found in other studies), but lower compared with age-matched ice hockey players (79%). Compared to the present study, they reported a lower prevalence of cam amongst male skiers (61% vs. 40%). Phillipon et al.’s study included a relatively small group of male skiers with closed growth plates and the study does not specify at which level or type of skiing (Mogul, Alpine, cross-country, etc.) the skiers train/compete.

Only few studies on females and cam morphology have been published, and there are no previous investigations on the prevalence of cam in female skiers. In other sports, female athletes have a reported lower prevalence of cam and this was also found in the present study [11]. We found that men had a threefold higher prevalence of cam morphology compared with females (61% vs 22%). Other studies have reported a higher prevalence of cam in women, but the methodology differs between the studies (radiographic method, cutoff for cam morphology, etc.). For example, Kapron et al. [21] found that 48% of female mixed athletes had cam morphology. Gerhardt et al. [13] found a prevalence of 50% cam morphology in elite female soccer players and 68% in elite male soccer players. On the other hand, in professional ballet dancers, Harris et al. [17] found that 12% of the female dancers had cam morphology, compared with 57% of the males.

In line with previous studies, we found that out of the seven measurements (from 9 o’clock to 3 o’clock), the largest *α*-angle was measured at 1 o’clock in the antero-superior region of the femoral head–neck junction [31, 40].

A strength with the present study is the equal distribution between females and males in the elite skiers group. More studies including female athletes are, however, needed to establish a greater understanding of cam morphology of the hips and the mechanism behind it. A highly relevant strength of the present study is that from early ages, boys and girls train together and are therefore exposed to a comparable amount of training, competing and load; i.e. this group of skiers train together depending on age and not sex. Regardless, the prevalence of cam morphology was shown to be considerably lower amongst the study’s female population. This could give important clues to the mechanism behind cam formation.

It is tempting to speculate if the difference in prevalence of cam morphology between females and males can be explained by the earlier closure of the female growth plate. When the load of training/competing increases with age, the growth plate is already closed and no cam morphology is therefore acquired.

The present study included both athletes and non-athletes of both sexes living and studying in the same geographical area. The non-athletes are a strength to this study and place the skiers results in perspective. The study includes a relatively large group of skiers that is equally divided between female and male skiers. A larger sample group with equal subgroup participation might have shown greater differences between the skiers and non-athletes, but also amongst the skiers divided into female/male and skiing disciplines. The inclusion criteria in the present study selected only a healthy population; however; this may have limited the ability to distinguish greater differences in the MRI measurements, compared with skiers presenting symptoms of FAIS.

## Conclusion

Young elite skiers are shown to have a higher prevalence of cam morphology of the hip, compared with non-athletes, and this appears to be more prevalent in males. This suggests that intense training load, during growth, may be a risk factor for the development of cam morphology in young elite skiers.
